# Neuro-toxoplasmosis and fatal necrotizing cerebellitis

**DOI:** 10.4322/acr.2021.363

**Published:** 2022-03-02

**Authors:** Gabriele Gaggero, Michela Campora, Beatrice Dose, Davide Taietti, Antonio Vena, Emanuele Delfino

**Affiliations:** 1 Ospedale Policlinico San Martino, IRCCS, Anatomic Pathology Unit, Genova, Italy; 2 Ospedale Santa Chiara, Division of Anatomical Pathology, Trento, Italy; 3 Università di Genova, Scuola di Scienze Mediche e Farmaceutiche, Department of Integrated Surgical and Diagnostic Sciences (DISC), Division of Anatomic Pathology, Genova, Italy; 4 Azienda Socio Sanitaria Territoriale (ASST) Ospedale Maggiore, Anatomic Pathology Unit, Crema, Italy; 5 Ospedale Policlinico San Martino, IRCCS, Infectious Diseases Unit, Genova, Italy

**Keywords:** Central Nervous System Infections, Central Nervous System Protozoal Infections, Toxoplasmosis, Cerebral, Cerebellar Diseases

Toxoplasma gondii is an obligate intracellular parasite (Protozoa family), generally hosted by cats and transmitted to humans by contact or eating undercooked infected meat. Infected individuals are usually asymptomatic, but immunocompromised persons may be severely affected: central nervous system (CNS) involvement by Toxoplasma is a typical complication of HIV/AIDS patients. It is the leading infectious cause of focal space-occupying lesions in the hemispheres and basal nuclei.^1^ However, a subtentorial toxoplasmosis with evolution into necrotizing cerebellitis is an exceedingly rare occurrence.^2^^,^ 3

The photos above refer to a 43-year-old South-American female presented to resume HIV antiretroviral therapy, voluntarily interrupted 3 years before. On admission, she showed headache and ataxia, arisen in the preceding days, a reason for further investigations. Bacterial and viral tests were negative, excluding HIV (1.3x10^6^ copies/mL on cerebrospinal fluid); however, the magnetic resonance (MR) revealed a cerebellar enhancing with leptomeningeal spread and mass effect compression of the 4^th^ ventricle and mesencephalic duct, which by anatomical location explains the neurological symptoms. MR features were compatible with an infectious hypothesis and other differential diagnoses (CNS lymphoma, glial neoplasms and demyelinating processes). The infectious hypothesis was clinically preferred, so intravenous broad-spectrum antibiotic therapy was administered: despite an initial benefit, the patient's neurological condition worsened again, and she died quickly before any further diagnostic/therapeutic steps could be taken.

**Figure 1:**
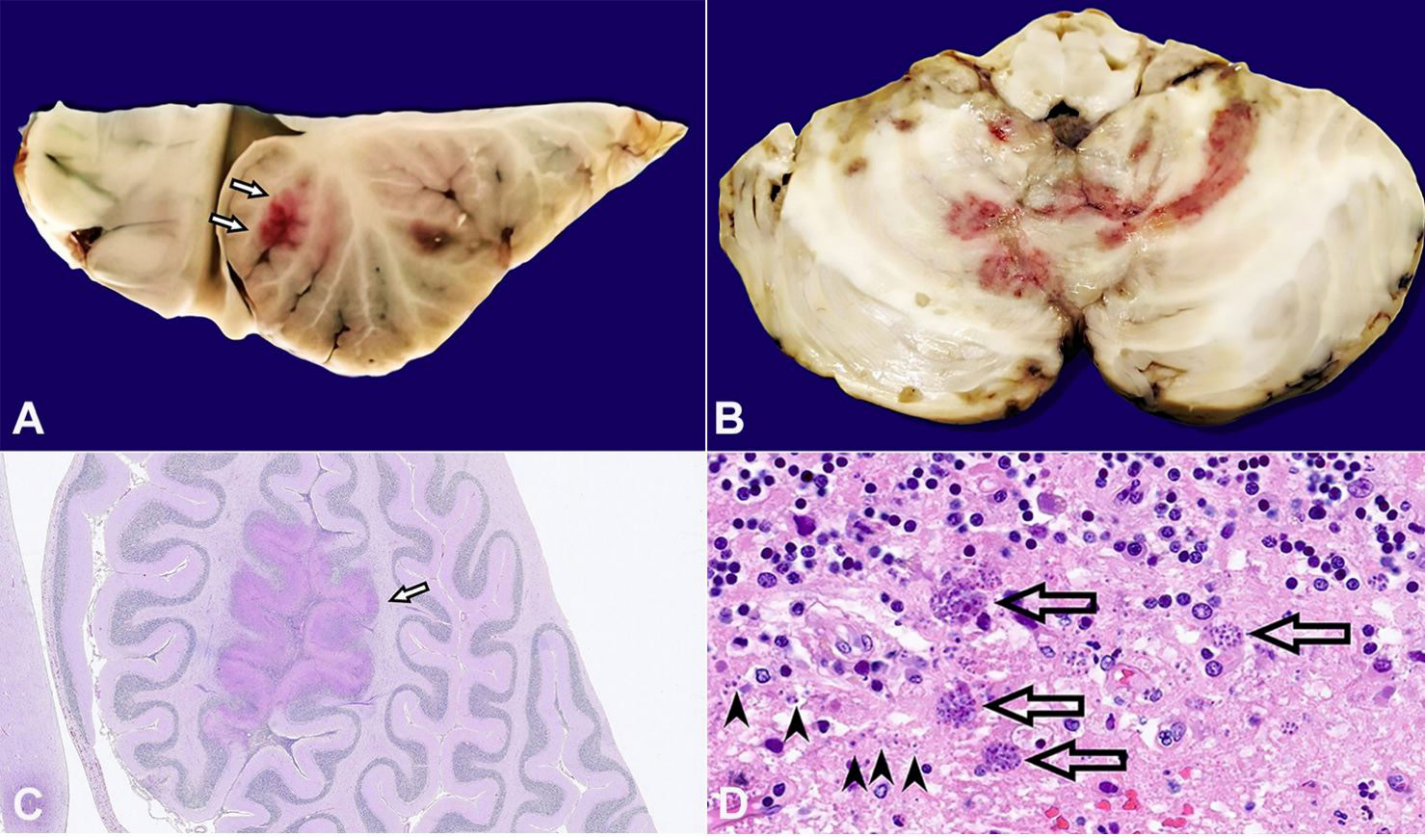
**A –** Cerebellar cutting (after formalin fixation): sagittal cut shows reddish friable area involving cerebellar folia (arrows); **B –** Axial cut shows reddish softened areas involving both cerebellar hemispheres and the cerebellar vermis; **C –** Microscopic view: at low magnification (Periodic Acid Schiff, magnification: 5x), a more intensely stained area is observed at the level of the cerebellar folia (arrow); **D –** Microscopic view: at higher magnification (H&E, magnification: 60x), this cerebellar area shows - at the transition between the granular layer and the molecular layer - the presence of necrosis and dark round corpuscles, consistent in size and morphology with Toxoplasma, both in the pseudocystic bradyzoite form (arrows) and in the dispersed/single tachyzoite form (arrowheads).

CNS examination, during the autopsy, revealed a dark red area on the cerebellar surface corresponding, at sagittal and horizontal cutting, to a 5 cm large reddish zone involving both cerebellar hemispheres and the vermis (Figures 1A and 1B). Histological examination confirmed the inflammatory/infectious nature of the lesion, excluding all the aforementioned hypotheses’ list. The presence of multiple abscesses with widespread parenchymal necrosis was observed, configuring a necrotizing cerebellitis (Figure 1C), with leukocytic inflammatory involvement also of the adjacent leptomeninges, confirming the MR findings; at higher magnification, multiple pseudocysts enclosing numerous hyperchromic corpuscles consistent with bradyzoites of Toxoplasma were observed at the transition between the cerebellar granular layer and the molecular layer. Single corpuscles dispersed in the parenchyma, consistent with the tachyzoite form of Toxoplasma, were also present (Figure 1D). From a strictly light microscopy point of view, such corpuscles lead to the differential diagnosis between three different protozoal infections: Malaria (Plasmodium falciparum), Toxoplasmosis (Toxoplasma gondii) and Trypanosomiasis (African trypanosomiasis by Trypanosoma brucei and South American trypanosomiasis by Trypanosoma cruzi, also known as Chagas' disease).^4^ Malaria can be ruled out because it requires the presence of corpuscles even in parasitized blood cells, which was absent, as were the necrotic/histiocytic intraparenchymal microfoci (so-called Durck's granulomas). On the other hand, Trypanosomiasis must be taken into consideration, not only for the corpuscles’ morphology, but also for the epidemiological data (patient’s South American origin) and for the Trypanosoma capability to cause necrotizing multifocal encephalitis in patients HIV/AIDS.^5^ However, it should be emphasized that CNS Trypanosoma cruzi infection usually requires the presence of amastigotes parasites in the glial and neuronal cells cytoplasms,^4^ which were instead histologically absent. PCR examination on paraffin-embedded tissue definitively confirmed it to be Toxoplasma.
